# Isolation of Aurantiamides from *Gomphrena Celosioides C*. Mart

**Published:** 2014

**Authors:** Omotayo Olutola Dosumu, Patricia Onocha, Olusegun Ekundayo, Muhammad Ali

**Affiliations:** a *Department of Chemistry, University of Ilorin, Ilorin.*; b *Department of Chemistry, University of Ibadan, Ibadan*^* . *^; c *H.E.J. Research Institute of Chemistry, International Center for Chemical Sciences, University of Karachi, Karachi-75270, Pakistan.*

**Keywords:** Amarantaceae, Gomphrena celosioides, Isolation, Aurantiamides

## Abstract

In West Africa and Nigeria in particular, many virgin plants are still waiting to be evaluated for their medicinal importance. Claims of plants with folk medicinal applications need to be evaluated and verified. *Gomphrena* *celosioides* (family – Amaranthaceae) is a weed grown in lawns and the biological activity of the extract had earlier been established. In the present study, the plant was collected, air dried, ground and soxhlet extracted with n-hexane and two compounds were isolated from the flakes that were recovered from the *n*-hexane extract on cooling. Column chromatography using 5% chloroform in n-hexane effected the separation. The structures of the isolated compounds were elucidated by spectroscopic analysis using IR, NMR (^1^H and ^13^C) and EI-MS. The compounds were found to be aurantiamide and aurantiamide acetate. This is the first report of isolation of these compounds in *Gomphrena* *celosioides*.

## Introduction

*Gomphrena* *celoisiodes* belong to the Amaranthaceae family which consists of about 120 species found in the Americas, Australia and Indo-Malaysia, 46 species in Brazil and with only few species in the forest in the Savanna vegetation, napeadic and high altitude grassland (1, 2). Many plants of this family are employed in folk medicine for their nutritive assets and treatment of several diseases (3, 4, 5). Brazil, in particular has employed a number of the species in the treatment of gastrointestinal and respiratory disorders as well as infectious diseases (6, 7). Previous phytochemical analyses of some species have led to isolation of hydrocarbons, alcohols, steroids, terpenoids, ecdysteroids, flavonoids, saponins, amino acids, butacyanins, reducing sugar and ketoses (2, 4, 7-13). 

Despite some phytochemical and biological activity studies which have been performed on certain species of the Amaranthaceae family, the genus *Gomphrena *still remains poorly studied. *Gomphrena* *celosioides* is a sprawling herb, native to Brazil, Paraguay, Uraguay and Argentina but has spread throughout the whole of the tropical world. In a study by Botha and Gerritsma-Vander (14), *Gomphrena celosioides* root extract exhibited weak antibacterial activity, while extracts of other parts were active on nervous autonomic system of rats with sympathetic and parasympathetic symptoms. In our previous biological evaluation of *G. celosioides* extracts, antimicrobial, anthelmintic and cytotoxic activities were established and also, 3-(4-hydroxyphenyl) methylpropenoate was isolated (15, 16).

In the present paper, the isolation and characterization of aurantiamide and its acetate form from the hexane extract of *Gomphrena celosioides* are reported and this is the first report of the isolation of these compounds from the plant.

## Results and Discussion

The dried whole plants were extracted by Soxhlet with *n*-hexane and this resulted in flaky crystals floating on top of the solvent on cooling. The floating crystals were collected and washed several times with n-hexane. The TLC of the dissolved crystals showed two spots which lead to column chromatographic separation on silica gel with 5% chloroform in n-hexane (see Experimental). This resulted in isolation of aurantiamide and its acetate.

*Aurantiamide (1)*

C_25_H_26_O_3_N_2 _showed a pseudo molecular ion peak at 384.3057 due to loss of water molecule. Actual molecular mass (M^+ ^402.0239, calc. 402.1804). It is a neutral compound with no characteristic UV absorption. Its IR spectra (KBr) showed the presence of amide carbonyl (3400 cm^-1^, 1650 and 1639 cm^-1^) and mono-substituted phenyl moieties (1570, 755 and 720 cm^-1^) (17). The ^1^H NMR (CDCl_3_) showed the presence of these groups with –CONH- signals at δ6.61 (^1^H, d, *J* = 7.5 Hz) and δ6.52 (^1^H, d, *J* = 7.5 Hz). The methylene group signals were between δ2.90 and 3.45. The 15 aromatic protons in a benzoyl (between δ7.48 – 7.68) with 3H producing multiplet and 2H producing doublet of doublet with *J* = 7, 2 Hz; Two benzyl of five protons in each group showing absorption at δ7.20 and 7.39, br. One pair of the methylene protons are magnetically equivalent while the others were not due to exhibition of conformational rigidity imposed on the molecule by intramolecular hydrogen bonding of the methylene group (17). The diasterotopic methylene protons signal adjacent to hydroxyl was observed at δ4.03 (^1^H, dd, *J *= 7.2, 6.4 Hz) and 4.50(^1^H, dd, *J *= 8.29, 5.38 Hz) (sub-structure (a)). The signals at δ4.61 (^1^H, m) and 4.19 (^1^H, dd, *J = *4.28, 4.03) were assigned to methine proton next to the diasterotopic methylene. 

The 100 MHz ^13^C NMR spectra showed the presence of two carbonyl (δ167.4 and 167.2) in addition to three methylenes (δ65.4, 37.6 and 37.3) and two methines (δ54.5 and 50.3). The δ65.4 was for primary alcohol and the homonuclear correlation between the diastereotropic protons adjacent to hydroxyl group at δ4.01 (δC 65.4, HMQC) with a multiplet (^1^H) at δ4.61 which in turn revealed a correlation with another diastereotropic benzylic proton at around δ2.90, thus confirming the sub-structure (a). Another benzylic diastereotropic proton at δ 3.20 was revealed by COSY to correlate with proton at δ 4.92 (H). The down field shift of this proton supports sub-structure (b). The presence of a benzoyl group was strongly supported by the ^m^/_z_ 105 in the MS, giving the sub-structure (c). 

**Figure 1:**
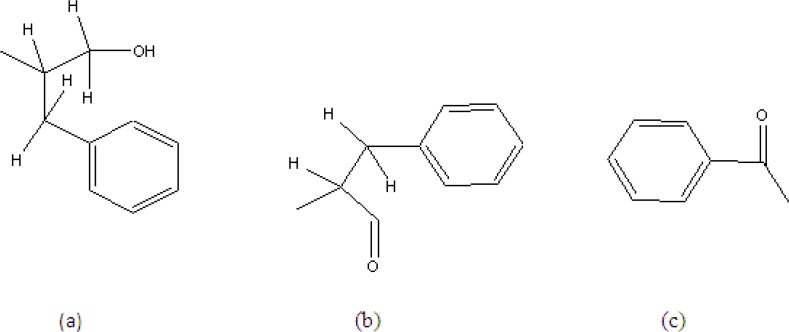
Sub-structure of aurantiamide

*Aurantiamide acetate (2)*

colourless needle like crystals with a molecular ion peak ^m^/_z _at 444.3016 for C_27_H_28_O_4_N_2 _ (calc; 444.2205) by mass spectrometry. Its IR spectra indicated the presence of an ester carbonyl group (1735 cm^-1^) and amide groups (3410, 1670, 1653 and 1645 cm^-1^), whose presence was also supported by two protons signals (δ6.73 and 5.96) in the ^1^H NMR and by two amide carbon signals (δ167.1 and 170.2) in the ^13^C NMR spectra. The homonuclear correlation 2D COSY (^1^H - ^1^H) experiments indicated the partial structures (d) and (e) together with unsubstituted benzoyl groups at δ7.23 - 7.70. The acetyl was also determined through singlet methyl at δH 2.02 ppm and its HMBC with carbonyl carbon δC 170.2 ppm. The presence of these unsubstituted benzoyl and benzyl groups were also confirmed by the intense fragment ions at ^m^/_z _105 (87%) and 91 (10%) respectively. The ^13^C NMR spectra of aurantiamide acetate gave 27 signals due to two amide carbons, acetyl carbons (δ20.8 and 170.7), 18 aromatic carbons, three methylene carbons (δ37.5, 38.4 and 64.6), two methine carbons (δ49.5 and 54.9). Signal assignments were confirmed by a 2D- long range CH correlation (COLOG) experiment (18).

**Figure 2:**
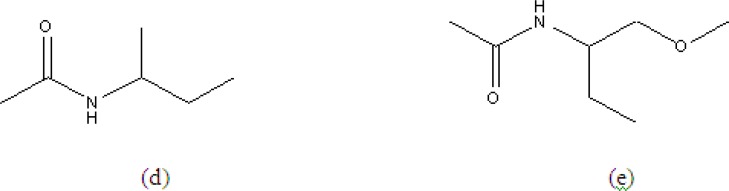
Sub-structure for aurantiamide acetate

The substitution of each group was confirmed by EI mass spectra, in which the fragment peaks at ^m^/_z_ 221 (21%) and 252 (35%) resulting in bond cleavage at either side of the central carbonyl group was observed. The above spectral data confirmed the structure of (2) as shown below. 

**Figure 3:**
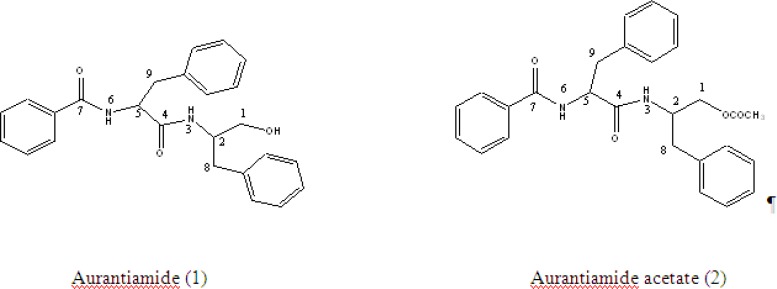
Structures of aurantiamide and aurantiamide acetate

## Experimental

*General methods*

IR spectra were recorded on a Pelkin Elmer spectrometer model 983 with KBr discs. The ^1^H (400 MHz), ^13^C (100 MHz) and 2D NMR spectra were recorded on Bruker WP200SY spectrometer. Chemical shifts, δ are in ppm with TMS as the internal standard. Coupling constants, *J* were given in Hz. MS was obtained at 70 eV using JOEL-MS route (Direct probe) and EI-MS were used for the mass spectroscopy.

*Plant material*

*Gomphrena* *celosioides* C. Mart was collected fresh from the premises of Abdulsalami Abubakar Graduate Hall, University of Ibadan, Nigeria. The identity was authenticated by Mr. Felix I. Usong of Forestry Research Institute of Nigeria, (FRIN) and Ibadan, where a specimen copy with voucher number FHI106429 was deposited. 

*Extraction and isolation*

Air-dried coarsely powdered whole plant (1.5 Kg) was percolated with hot n-hexane (5 L) for 12 h at regulated temperature of 40-50 ^o^C. Whitish flakes were obtained floating on the n-hexane extract after cooling. The white flakes were collected and washed several times with n-hexane. The crystals were dissolved, spotted and developed on TLC. TLC on n-hexane/CHCl_3_ showed two spots. Column chromatographic separation effected with 5% CHCl_3_ in n-hexane afforded compounds 1 (12 mg) and 2 (17 mg).

*Aurantiamide (1)*

White amorphous solid, mp 186-188 ^o^C. 

IR (KBr) _max _cm^-1^: 3440 (OH, NH), 1639 (-CON), 722 (monosubstituted phenyl).

^1^H NMR (400 MHz, CDCl_3_), (ppm): 6.61 (^1^H, d, *J *= 7.50 Hz, H-3), 6.51 (1H, d, *J *= 7.50 Hz, H-6), 4.03 (^1^H, dd, *J =* 7.21, 6.44 Hz, H-1a), 4.05 (^1^H, dd, *J *= 8.29, 5.38 Hz, H-1b), 4.61 (1H, m, H-2), 4.19 (1H, dd, *J *= 4.28, 4.03 Hz, H-5), 3.20 (2H, m, H-8), 2.91 (2H, m, H-9), 7.20 – 7.68(15H, m, Ar-H). 

^13^C NMR (100 MHz, CDCl_3_), (ppm): 65.4(C-1), 50.3(C-2), 167.4(C-4), 54.5(C-5), 167.2(C-7), 37.3(C-8), 37.6(C-9), 126.8-137.2 (Ar-C). 

EI-MS: ^m^/_z_ (rel. int.): 77(14) [C_6_H_5_]^+^, 91(17) [C_7_H_7_]^+^, 105(87) [C_6_H_5_CO]^+^, 129(5) [C_6_H_5_CH = NH_2_]^+^, 131(8) [C_6_H_5_CH = CHCO]^+^, 224(47) [C_6_H_5_CO-NH-CHCH_2_Ph]^+^, 252(72) [C_6_H_5_CONHCH(CO)CH_2_C_6_H_5_]^+^, 293(12) [M-(MeCO_2_H + C_6_H_5_CH_2_)]^+^, 353(4) [M-C_6_H_5_CH]^+^, 384(7) [M-H_2_O]^+^. The fragments ^m^/_z_ 134(4) [C_6_H_5_CH_2_CH = CHOH]^+^, and 311(8) [M-C_6_H_5_CH_2_]^+^, are indicative of the presence of the corresponding amide. 

*Aurantiamide acetate (2)*

colourless needle like crystal, mp 183-184 ^o^C (uncorr.)

IR (KBr) _max _(cm^-1^): 3440 (NH), 1735 (CO_2_), 1639 (NHC = O)

^1^H NMR (400 MHz, CDCl_3_), (ppm): 2.05 (3H, s, H-2”), 3.04 (^1^H, dd, *J* = 8.55, 13.68, H-8), 3.18 (1H,dd, *J* = 5.99, 13.68, H-9), 3.81 (^1^H,dd, *J* = 4.27, 11.12, H-1b), 3.91 (^1^H, dd, *J* = 5.13, 11.12, H-1a), 4.74 (^1^H, ddd, *J* = 5.99, 8.55, = 7.68, H-2), 5.96 (1H, d, *J* = 8.55, H-3), 6.73 (1H, d, *J* = 7.69, H-6), 7.05-7.69 (15H, m, Ar-H).

^13^C NMR (100 MHz, CDCl_3_), (ppm): 37.5 (C-1), 49.5 (C-2), 169.2 (C-4), 48.4 (C-5), 170.7 (C-7), 37.3(C-8), 37.6(C-9), 174.2 (C-1”), 20.7(C-2”), 126.7 - 157.1 (Ar-C). 

EI-MS ^m^/_z_ (rel. int.): 76.9(27.7) [C_6_H_5_]^+^, 91(10.4) [C_7_H_7_]^+^, 105(89) [C_6_H_5_CO]^+^, 120(3.5) [C_6_H_5_CH = NH_2_]^+^, 131(5.5) [C_6_H_5_CH = CHCO]^+^, 224(17) [PhCONHCHCH_2_Ph]^+^, 252(19) [224 + CO]^+^.

## Conclusions

*Gomphrena celosioides* contain aurantiamides and its acetate. These compounds have proved to be active against microorganism even at very low concentrations (19). This finding has supported the use of this plant in folk medicine for treatment of gastrointestinal, respiratory or infectious diseases. Trial test of these compounds for anti-tumor and insecticidal property should be studied since the plant extracts has earlier shown positive cytotoxicity against brine shrimp (16). 
